# A Review of Swine Transportation Research on Priority Welfare Issues: A Canadian Perspective

**DOI:** 10.3389/fvets.2019.00036

**Published:** 2019-02-22

**Authors:** Fiona C. Rioja-Lang, Jennifer A. Brown, Egan J. Brockhoff, Luigi Faucitano

**Affiliations:** ^1^The Royal (Dick) School of Veterinary Studies, University of Edinburgh, Edinburgh, United Kingdom; ^2^Prairie Swine Centre, Saskatoon, SK, Canada; ^3^Department of Animal and Poultry Science, University of Saskatchewan, Saskatoon, SK, Canada; ^4^Prairie Swine Health Services, Red Deer, AB, Canada; ^5^Agriculture and Agri-Food Canada, Sherbrooke R&D Centre, Sherbrooke, QC, Canada

**Keywords:** animal welfare, carcass quality, meat quality, pigs, stress, transport

## Abstract

The purpose of this review is to present the best available scientific knowledge on key animal welfare issues during swine transport, such as transport duration and distance, time off feed and water, rest intervals, environmental conditions, loading density, and transport of young animals, based on their impact on stress, injury, fatigue, dehydration, body temperature, mortality, and carcass and meat quality. The review was limited to this set of priority welfare issues which were identified by the National Farm Animal Care Council (NFACC) Scientific Committee to help with the development of the livestock transportation Codes of Practice. This review focuses primarily on research related to the transport of market pigs (100–135 kg) which is a reflection of the current literature available on pig transportation. This information presented here can be used to support other animal welfare codes, guidelines, standards or legislations regulating the welfare of pigs during transport. Based on the available literature, clear conclusions can be drawn on the impact of vehicle design, pre-transport fasting, control of environmental conditions and loading density on the welfare of pigs during transport and on pork quality. However, the effects of journey duration are still unclear and a recommendation on the maximum transport time cannot be provided. Further studies investigating the impact of factors, such as ambient conditions within the transport vehicle, loading density at extreme ambient conditions, travel distances, maximum travel duration, rest/stop duration, and management of pigs during rest stops are required. More specifically, further research in relation to the welfare of market weight, newly weaned and breeding pigs, and cull sows and boars during transport is needed.

## Introduction

Pigs in Canada are usually transported at least once in their life, either as young piglets, when transferred to grow-finish facilities, or as older pigs when being sent for slaughter. Gilts and boars are also transported from genetic nucleus sites to commercial farms (1). The welfare of pigs during transportation depends on many interacting factors, such as the condition of the animal at time of loading, ambient temperature, loading density, time in transit, social stress (e.g., mixing with unfamiliar pigs), handling, unfamiliar noises and smells, vibrations, and sudden speed changes (2, 3). These factors are potentially stressful and, in combination can also have a significant impact on the pigs' physiology, resulting in meat quality defects at slaughter. The term stress is used frequently throughout this review as a way of suggesting negative implications (defined as acute or chronic stress) on pig welfare during transportation.

Loading is generally considered the most critical stage of the transport period, mostly in terms of physical and psychological challenge, as shown by increases in heart rate (4, 5), body temperature (6, 7), and blood cortisol and lactate values (8–10). These responses to transport stress are not only indicators of reduced welfare but may also have an effect on *peri-mortem* muscle metabolism and thereby on meat quality. Stress at loading can result from factors, such as mixing unfamiliar pigs, distance moved from the pen to the loading point, group size, handling system, design of the alleys, light and sound, the handling skills of personnel, and design of the loading device [either ramp or quay/dock; (11)]. Vehicle design features, such as the loading system (ramps or hydraulic platform), microclimate control, and floor type can also impact the welfare of pigs during transport (12).

**Figure 1:**
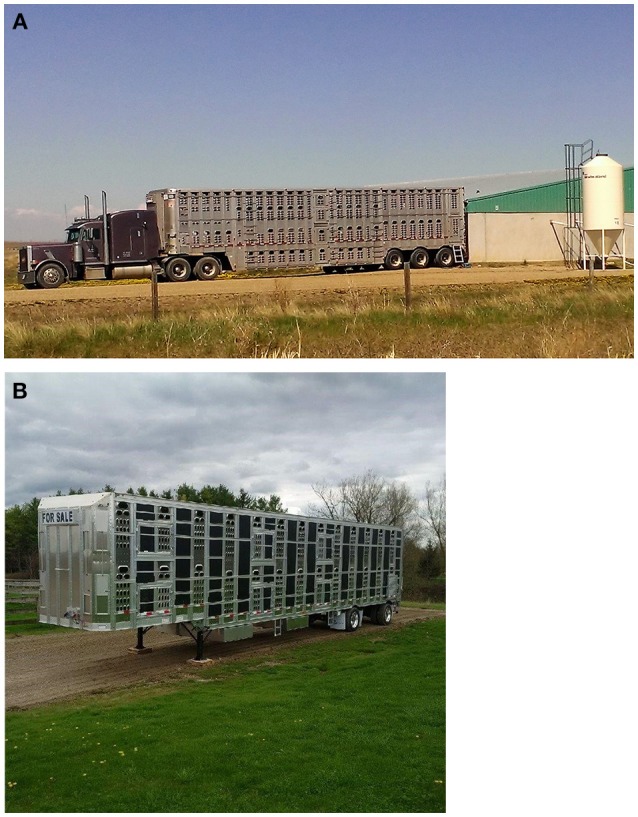
Types of vehicles used in Canada: **(A)** “pot belly”; and **(B)** flat deck trailers (courtesy of E. Brockhoff, Prairie Swine Health Services, Canada, and A. Hurst, Luckhart Transport Ltd., Canada).

**Figure 2:**
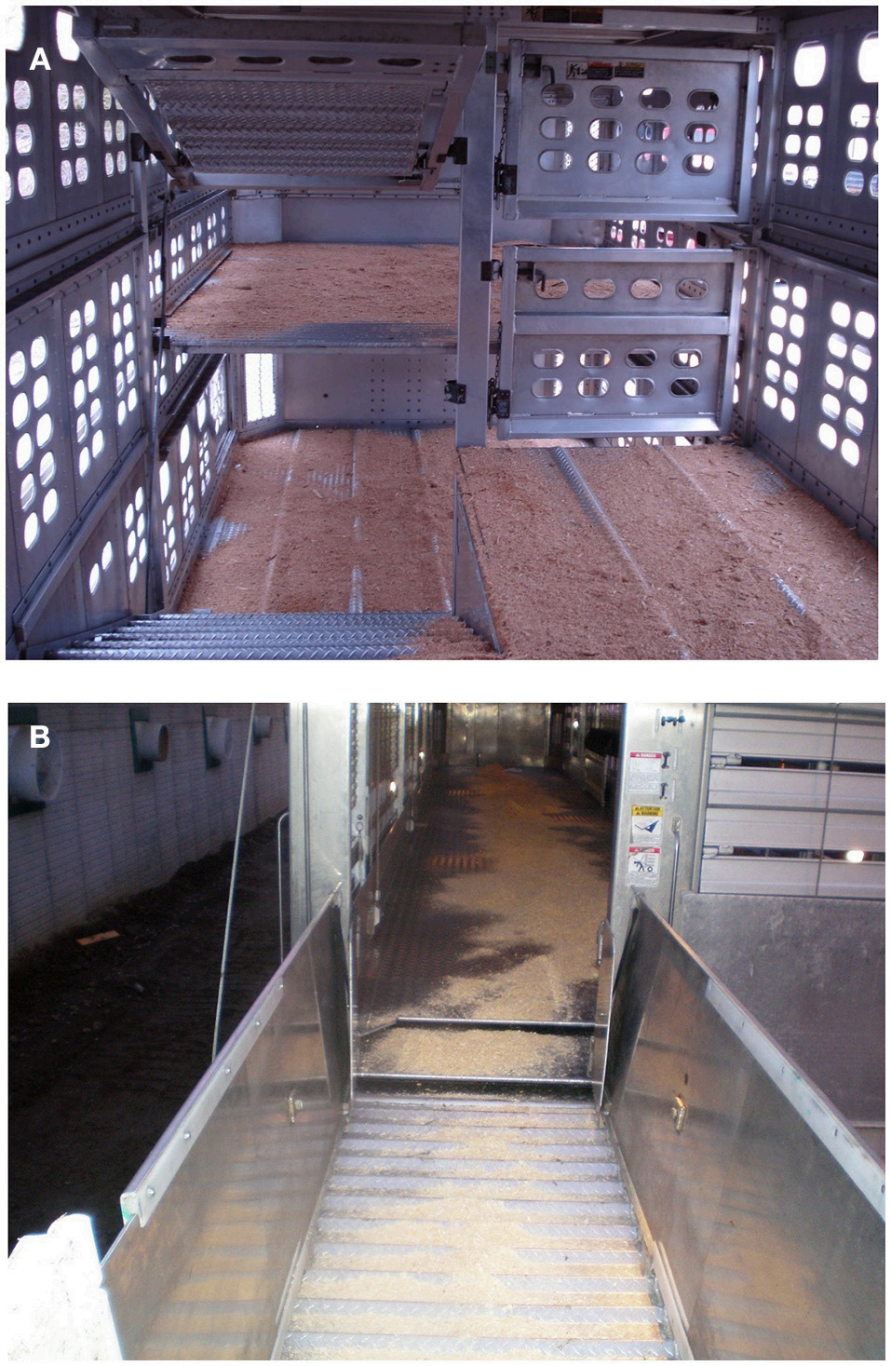
Inside view of: **(A)** “pot belly”; and **(B)** flat deck trailer (Dr Luigi Faucitano, Agriculture and Agri-Food Canada).

The types of vehicles used for pig transportation in Canada vary from small single-deck trucks to large three-deck punch-hole trailers, with either “pot-belly” or straight/flat-deck designs (Figures 1A,B, 2A,B). Pot-belly trailers are widely used as they are versatile and can transport large loads (up to 230 slaughter-weight pigs) in a single journey (5, 13). However, they have been criticized because of difficulties in handling pigs due to the need to negotiate multiple internal ramps and turns (14, 15) and poor internal climate conditions (16–19). These internal conditions can either result in a higher percentage of pigs showing open-mouth breathing and skin discoloration at unloading, or greater animal losses and poor pork quality when compared with other trailer designs (20).

## Methods

The objective of this review was to collect and synthesize the results of peer-reviewed literature related to the welfare of pigs during transportation, specifically with a Canadian perspective. The review was limited to a set of priority welfare issues which were decided on by the National Farm Animal Care Council (NFACC) Scientific Committee, as NFACC provided the financial support for this work. This review will go on to support the development of the NFACC Codes of Practice for the transportation of livestock. The priority welfare issues identified by the Scientific Committee and other stakeholders specifically focused on the effect of transport duration, time off feed and water, rest intervals (where appropriate by species), environmental conditions, and loading density, as single factors or in combination, on animal welfare. The review, where possible, identifies measures to mitigate the impact of environmental conditions. The main databases used in the preparation of this report were CAB International, Scopus, and Science Direct. There were no limits set on the publication date of the articles used. Mostly, peer reviewed resources were evaluated for inclusion in the review, and in some instances industry publications were included.

This review focuses primarily on research related to the transport of market pigs (100–135 kg) which most research on pig transportation has focused on. The reviewed literature results are largely based on field studies. However, some research on weaners was conducted using simulated transport. There is little literature available that specifically addresses the transportation of cull sows, boars, and newly weaned piglets. However, pigs at other stages of production may need special considerations during transport, due to their physiological conditions, health status, or age (21). For example, sows are most likely to be culled due to lameness or failure to rebreed (22, 23), and may have difficulties walking, and loading onto the truck. Many cull sows also have poor body condition (24), whereas obesity has been indicated as the main reason for culling boars (25) along with feet and leg issues (26). Peterson et al. (27) reported that for cull sows the risk of death during transport was 1.93 and 0.81 times higher at outdoor ambient temperatures ranging from 29 to 33°C and from 4 to 10°C, respectively, compared to 12 to 26°C.

## Transport Duration and Distance

In Canada, the large expanse of the territory coupled with the consolidation of the slaughter industry results in pigs being transported for long distances and durations [>7 h; (2, 6, 17)].

Indicators of poor welfare have been reported in slaughter pigs for both long and short journeys (28, 29). Quoting Warriss (30) “A short journey under poor conditions may compromise welfare as much as, or even more than, a long journey under good conditions.” Some studies present evidence that shorter transport distances (<100 km) may be more detrimental resulting in a higher number of dead on arrival (DOA) than longer ones as the stress of loading and unloading over a short period of time is compounded. In contrast, on long journeys under suitable conditions, pigs are able to recover from the stress of loading and have time to acclimate to transport before unloading (10, 31–36). It has been observed that pigs increasingly began to sit and lie down after 20–30 min of transport, indicating that pigs are more vulnerable to fall or be thrown around due to vehicle movements during the initial period of transport when they are more likely to be standing up (37).

Haley et al. (29) found that for every 50 km increase in distance, transport mortality decreases by 0.03%, and in-transit death losses were lower for transport distances >135 km. However, in this study there were large differences in mortality risk between producers (94% of the producers had no deaths). It is possible that there were confounding factors between producers and distance to the slaughter plants (i.e., some of the producers with high mortality may have been located closer to the slaughter plants than those with a lower mortality). Thus, to confirm these results, controlled studies, where the farm (or herd) and travel distance factors are blocked, are needed. Rearing conditions and pre-transport management also accounted for some of the difference in DOA between producers. Sutherland et al. (36) reported a positive linear relationship between mortality risk for pigs transported to slaughter in the USA and increases in journey duration from 0.5 to 4 h. Although, they reported that the risk then decreased as journey duration increased from 5 to 10 h, the regression coefficient was positive rather than negative, suggesting that the mortality risk actually increased with journey duration. Averós et al. (38) used multivariable analyses to identify risk factors for mortality of pigs transported to slaughter in the EU. There was an interaction between the duration of pre-transport fasting and journey duration. For journeys up to 8 h in pigs that had not been fasted, the risk of mortality increased with journey duration, but in those that had been fasted, there was no effect of journey duration (of up to 24 h) on mortality risk.

Short journeys (2 h and less) result in increased concentrations of cortisol and lactate in exsanguination blood, resulting in higher risk of pale, soft and exudative (PSE) pork (39, 40), and in pigs being more difficult to handle at the slaughter plant (41). During short transportation trials (45 min), using both pot-belly and flat-deck trailers, Weschenfelder et al. (18) reported an increased level of fatigue (based on exsanguination blood lactate level) at the time of slaughter in pigs hauled with the pot-belly trailer. The authors concluded that the pigs transported for such a short time did not have sufficient time to recover from the stress of loading in the pot-belly trailer due to internal ramps and turns.

However, there is also evidence that pigs transported for long durations (>16 h) may be more exposed to fatigue and dehydration, as shown by the higher blood glucose, lactate and hematocrit levels at slaughter (42–44). When compared to 6 and 12 h of transport in winter, Goumon et al. (6) and Sommavilla et al. (45) reported that pigs, which were transported for 18 h, had greater gastrointestinal tract temperatures, higher exsanguination blood CK levels, and drank more water and took longer to rest in the lairage pen. In a study investigating the transportation of fattening pigs in Mexico, Mota-Rojas et al. (43) transported fattening pigs for 8, 16, and 24 h without access to feed or water. The authors reported increased incidence of bruising, redness of the skin, muscle tremors, and number of pigs lying down upon arrival at the slaughter plant in pigs transported for the longest time (24 h).

However, it is likely that the additive effects of vehicle design, fasting duration, mixing, ambient, and transport conditions, and pig genetics make significant contributions to the relationships between journey duration and risk of fatigue and exhaustion of muscle glycogen stores (17, 18, 46–49).

There are few studies in the literature which focus on the effects of transport duration on newly weaned pigs or breeding pigs. Sutherland et al. (50) assessed the effects of 0, 6, 12, 18, 24, or 30 h of transportation on the well-being of breeding-age gilts using multiple indices of stress (including granulocyte to lymphocyte ratio (G:L), blood cortisol level, metabolic homeostasis, muscle exertion, and reproductive performance) under USA transport conditions. In this study, non-transported gilts (control) remained in their home pen and had access to food and water during the entire experimental period. The study found that gilts transported up to 30 h experienced acute stress during the initial 6 to 12 h, while having changes in water homeostasis throughout the 30-h journey due to dehydration and food deprivation. The G:L ratio was greater in the transported gilts after 6, 12, and 18 h of transportation than in control (non-transported) gilts. Blood cortisol concentrations were also greater among the transported gilts after 6 h compared with non-transported gilts. In animals transported for 12 and 30 h, blood cortisol, G:L ratio, and cytokine levels were all within baseline levels. However, an increase in blood albumin and total protein concentrations suggests that pigs were experiencing dehydration.

Overall, there appears to be a growing body of literature that supports the view that short as well as long transportation times can be detrimental to animal welfare, although the information about animal losses, including dead and non-ambulatory pigs on arrival at the plant, was not recorded in most reviewed studies (6, 17, 18, 39, 40, 42–45, 50). The length of journey does not appear to be the most important factor in terms of pigs' response to transport; other transport factors (e.g., weather, driving technique, stress susceptibility, vehicle design, location within the truck, and pig health) also play an important role in the animals' response (6, 17, 18, 31, 47, 51). However, longer duration transports have the added limiting factor of prolonged time off feed and water, especially considering that fasted pigs must rely on body energy reserves to survive and cope with transport and handling stress.

## Time off Feed and Water

It is generally recommended that slaughter pigs are taken off feed as part of the on-farm preparation before transport (52, 53). This practice results in fewer animal losses (54, 55), especially in hot weather conditions and in stress-susceptible pigs (31). Fasting also reduces travel sickness (10, 56), as shown by the decreased circulating levels of vasopressin during transport compared to pigs that were not fasted (57).

The death of unfasted pigs during transport can result from the pressure of the full stomach on the vena cava, resulting in decreased blood flow efficiency (58). However, it has also been reported that groups of pigs fasted 18 h prior to loading may be more difficult to handle at loading as shown by the greater proportion of pigs going backwards, making 180° turns, and vocalizing (59). These behaviors are a possible reflection of increased frustration, fatigue, and excitement caused by hunger (60, 61).

It is reported that pigs will lose approximately 4% of body weight during the first 18 to 24 h of the fasting interval (57). A study by Brumm et al. (62) investigated the effects of out-of-feed events in grow-finish pigs and reported that when pigs omit one or more meals in a 24 h period, they are unable to compensate for this. Similarly, when feed is withdrawn for more than 24 h it is likely to result in catabolism of body stores (3). Lambooij (3) also states that liver glycogen is completely depleted after 12 and 18 h of food deprivation at the slaughterhouse, with live weight loss decreasing by approximately 0.21%/h. However, Dantzer (63) stated that in fasting for up to 24 h, the loss of live weight and carcass weight mainly results from excretion, evaporation, and respiratory exchange, which are normal bodily functions. Only after 24 h of fasting, real body weight losses occur at a rate of 100 g of weight loss per additional hour (64). In light of these findings, Faucitano et al. (64) have suggested that a fasting interval of 16 to 24 h might provide an optimal compromise between animal welfare, food safety, and meat quality.

Extending the fasting interval (up to 72 h) results in physiological and behavioral changes, such as reduced blood glucose levels (65), increased fighting rate in mixed groups due to hunger-related irritability and excitement (43, 59, 66, 67), and increased drinking rate (6, 43, 64), as responses of pigs to maintain their homeostasis.

When pigs are denied access to water (or are unable to access it), additional weight loss will occur due to dehydration even during short journeys (31). Factors that contribute to an increased rate of dehydration during transport are increased ambient temperature, decreased humidity in the compartment, and increased airflow and body temperature (31). Dehydration also causes a loss of muscle tissue (which is composed of about 75% water; 31). However, it has also been observed that even when water is available on the trailer, pigs consume little to no water due to the lack of space and poor stability during vehicle movements (68, 69). Even at rest stops, water intake may be limited if pigs are unfamiliar with the type of nipple drinker, or cannot get access to the drinker due to other pigs blocking or using the drinker.

Based on the current data, pre-transport fasting should be applied to reduce animal losses and travel sickness in market pigs during transport compared to pigs that are not fasted. However, total fasting time (from last meal to slaughter) should not be too long to avoid carcass yield losses, aggressiveness and dehydration.

## Rest Intervals

When the allowed maximum travel time is achieved (8 to 36 h depending on the region of the world; 2, 11), pigs are unloaded from the truck and walked to pens where they are fed, watered, and rested. Rest intervals are intended to allow pigs to recover from the effects of dehydration, hunger, and general fatigue before being reloaded onto the truck to continue their journey. However, the stress of unloading and loading animals at a rest stop combined with mixing in a novel environment may be detrimental to the pigs' welfare (2).

A less stressful practice may be to feed and water pigs on the truck, as this will avoid the stress of unloading, reloading, and mixing (70). However, in a study comparing the effects of keeping market pigs on the truck vs. in a rest stop during a 9-h rest period after 20 h of transport, Chevillon et al. (71) found higher heart rates in pigs at unloading and reloading at the rest stop, but no differences in resting behavior, feeding and drinking rates, weight loss, or carcass yield between the two practices. Furthermore, off-loading of animals at a common site poses a significant risk to biosecurity and cross-contamination between loads (71).

This discrepancy in international standards is due to the fact that a minimum resting time after long transportation is not yet established (2). Research is also needed to validate good practices aimed at minimizing the stress of handling animals for rest periods during transport.

## Environmental Conditions

Pigs transported in Canada can experience extreme temperature fluctuations, often falling below or exceeding their thermal comfort zone (10 to 24°C; 53). Various Canadian swine transport trials conducted over a 10 years period reported ambient outdoor temperatures ranging from −28.8 to 1.9°C in the winter season and from 9.1 to 40.1°C in summer (5, 6, 13, 16, 19, 72).

Extreme environmental temperatures during transit are generally considered to be one of the greatest contributors to transport losses in terms of pigs dying (29, 36, 73). Pigs do not sweat; therefore, they are limited in their ability to thermoregulate in hot environments and are sensitive to heat stress (74). As ambient temperature increases, pigs modify their behavior to reduce heat production by reducing activity (75, 76) and also dissipate heat by accelerated breathing and increasing contact with cool or moist surfaces (20, 77, 78). However, at temperatures >30°C with relative humidity levels >88% these evaporative cooling mechanisms are significantly compromised (30, 31, 79). Under cold conditions, they will change posture and huddle together to maintain body temperature and limit heat loss (80). Some of these behavioral changes to cope with hot and cold conditions may be difficult to achieve within the confined space of a transport vehicle.

The thermoneutral zone for pigs during transport is dependent on many factors including their size, duration of fasting, floor type, air velocity, and group size. For example, the thermoneutral zone varies across pig weights: 2 kg: 31 to 33°C; 20 kg: 26 to 33°C; 60 kg: 24 to 32°C; and 100 kg: 23 to 32°C. If the pigs are able to lie on a well-bedded surface, the lower critical temperatures can be reduced by 3 to 5°C (81).

Interactions can occur between journey duration, external temperature, and pig type (weaner or market weight pigs) that affect the risk of mortality during transport. Zhao et al. (79) tried to examine the effects of the relationship between journey distance (<600 km; 600 to 900 km; 900 to 1,200 km; 1,200 to 1,500 km; and >1,500 km) and ambient temperature (<15°C or cool/cold; 15 to 25°C or mild; and >25°C or warm/hot) on mortality rate in weaned and slaughter pigs. To this end, a total of 7,056 transportation records of weaned pigs (3,174 records) and slaughter pigs (3,882 records) for the period from April 2012 to January 2014 were provided by a US swine company. For weaned pigs transported at external temperatures <15 °C, the mortality risk was lower at journey distances <900 km than at >900 km. Meanwhile, the mortality risk increased in weaned pigs transported at >25° with journey distances <600 km and >1,500 km. For market weight pigs transported at <15–25°, journey distance had no effect on mortality, but in those transported at >25°, the mortality risk was greater during journey distances of 1,200 to 1,500 km than during shorter journeys (79). This study provides some helpful insights into the relationships between journey duration and ambient temperature; however, it should also be noted that the results may have been confounded by other uncontrolled factors, such as pre-transport management of the pigs at the finishing farms.

## Extreme Temperatures—Heat

Under natural conditions pigs would wallow to thermoregulate, a behavior which is not possible during transportation. Heat stress is caused by the interaction of environmental factors, including temperature, air velocity, and humidity, and may not only cause a decrease in body weight, but may also affect well-being (3). The frequency of heat stress indicators (e.g., panting, skin discoloration) has been shown to increase in warmer months (20). Haley et al. (28) also reported that when temperatures inside the vehicle increased, death losses also increased. In-transit mortality increase at ambient temperatures of ≥20°C for market weight pigs (82–84). More precisely, the risk of death for market-weight pigs during transport can be 1.4 times higher at temperatures between 29 and 33°C than in a temperature range of 12–26°C (27).

Although pigs lack functional sweat glands that respond to high ambient temperature, evaporative heat loss is a major way for pigs to lose heat. Evaporative heat loss can be increased by increasing respiration rate and evaporation of water from wetted skin (e.g., achieved by misting pigs). In conditions of high temperature and high humidity, respiratory evaporation is impaired so any heat loss from evaporation of water from wet skin is beneficial (85). Modern pigs may have less ability to withstand high temperatures during transport compared with pigs that were raised several decades ago. Indeed, the rapid growth of modern market pigs combined with a small heart size relative to body mass and acute stress during transport can result in tachycardia and death due to heart failure (86). Recent evidence also suggests that pigs may suffer from heart abnormalities, which predispose them to cardiac failure (87).

Pigs transported in hot conditions also display a variety of behavioral and physiological changes. Kephart et al. (88) reported that the number of pigs that arrive at the slaughter plant showing open-mouth breathing increases at ambient temperatures >17°C. Pigs are also more likely to lie down during transport in summer months (6, 15, 89) either due to heat exhaustion or in attempts to maximize heat loss through contact with the truck surfaces. Some compartments within passively ventilated trailers are prone to higher temperatures both under moving and stationary conditions leading to increased physiological stress for pigs (16–19). For example, Conte et al. (7) showed that in pot-belly trailers the top front, rear top, and bottom rear compartments were warmer and required greater effort at loading due to the steep internal ramps of up to 32°C used to enter these areas. Pigs loaded in these compartments in Canadian summer months showed increased gastro-intestinal tract temperatures (GTT) after loading and during transport compared to other compartments (7). Similarly, extreme pig surface temperatures were recorded in the bottom rear and top middle of US double-decked trailer (90). The microclimate in these compartments, combined with the additional physical effort required to negotiate the steep internal ramps has been suggested to cause poor meat quality compared with meat from pigs transported in other compartments (5, 13). Furthermore, Haley et al. (29) and Correa et al. (5) reported greater animal losses in summer. Haley et al. (29) reported the highest number of deaths being recorded during the month of August (0.4%) when the maximum ambient temperature was 33.6°C.

## Extreme Temperatures—Cold

Canadian transport studies have reported that market pigs hauled in winter were more difficult to handle at loading and unloading (14, 15), spent more time standing during transport (6, 14, 15), and had higher heart rates during transport and unloading (6, 13). Furthermore, pigs transported in winter spent more time drinking in lairage and had more carcass bruises (6, 48). In US studies, Sutherland et al. (36) found increased percentages of non-ambulatory pigs on arrival at the slaughter plant when ambient temperatures were below 5°C. Guàrdia et al. (54) similarly reported greater losses of pigs in winter (0.27%) when recording monthly mortality rates in 16 Spanish abattoirs. In the above-mentioned retrospective study on ambient conditions and transport losses, Peterson et al. (27) found that the risk of death was 0.97 times greater in hauls occurring at 4–10°C than at 12–26°C. The explanation for the greater animal losses in colder months compared with summer can be the result of the extra care taken in summer, such as no mixing of unfamiliar pigs, showering in transport and lairage, and night transportation to reduce the effects of heat stress (54) or cold stress, heavier market weight, increased load size, and changes in health status (91), while no such measures were implemented during winter months (54).

## Measures to Mitigate the Impact of Environmental Conditions

Several practices have been developed and studied to reduce and mitigate the impact of environmental challenges, including proper use of bedding, ventilation, misting and sprinkling systems, and adjusting space allowance (see following section). The amount and type of bedding material used in trailers can be adjusted according to season. In summer, transporters are encouraged not to overuse bedding as this may increase pig losses (92). Commercial transporters use either wood shavings or straw in winter. Straw provides greater insulation and is also easier to remove than shavings when frozen (93).

In a study examining the use of bedding on trailers in each season, the authors reported that with increasing bedding used in summer (wood shavings, with either 3, 5, 7, or 9 bales/trailer) the rate of dead and down pigs increased in a linear manner (92). In winter, additional dry bedding is recommended to help insulate the pigs and maintain their body temperature (94). Indeed, when bedding is insufficient, frostbite can occur on the pigs' skin as anecdotally reported by Goumon et al. (6) in winter transport studies. These injuries result from insufficient protection between the pigs' skin and the metal truck floor (insufficient bedding), or through prolonged contact with the outside air via perforations in the trailer (e.g., due to overcrowding). In winter, the thermal properties of the trailer can also be modified to reduce heat loss. The use of Styrofoam insulation (95) and polyester floor type (96) were shown to increase internal truck temperatures during transport under cold conditions (−20°C) and to improve pork quality, respectively.

In high ambient temperatures, ventilation rates on trucks used in Canada can be increased, either by opening side perforations to allow the air to freely circulate, or by active ventilation, through the use of fans. In passively ventilated vehicles, like most North American trailers, the most common method of ventilating compartments is via vents positioned at the upper part of the left and right sides (3). The opening type, punch or slatted, can also make a difference in the air-flow inside the vehicle during movement (17). However, when the vehicle stops during a journey, the lack of air circulation leads to a rapid increase in internal temperatures at a rate of approx. 1°C temperature rise per minute up to 3–4°C rise in 5 min (90), with the bottom front compartments being up to 10°C warmer than the external ambient temperature during the stop (16–19). These higher ambient temperatures are more likely to lead to pig losses.

In stationary trailers, pigs can be cooled by active (fan) ventilation, water sprinkling, or a combination of ventilation and water sprinkling (evaporative cooling). Water sprinkling/misting (97) and active ventilation (98, 99) in a stationary truck have been shown to reduce deaths during transport. Colleu and Chevillon (98) found that sprinkling pigs at ambient temperatures >10°C in one deck of a trailer helped to reduce skin temperature by 10%, compared to non-sprinkled pigs on another deck in the same trailer. A more recent Canadian study compared trailers with and without sprinkling. It was found that when ambient temperatures exceeded 23°, the application of 5 min of water-sprinkling just prior to leaving the farm and immediately before unloading at the slaughter plant reduced drinking behavior in lairage compared with unsprinkled pigs (19). In this study, core body temperature tended to be lower in sprinkled pigs, which may explain the reduced need to drink water on arrival in the lairage pen. When sprinkling was applied at ambient temperatures of 20°C and greater, it reduced exsanguination blood lactate concentration, an indicator of fatigue, and meat exudation in pigs transported in the middle front and rear compartments, compared with pigs in the same compartments of an unsprinkled trailer (100).

However, water sprinkling with insufficient ventilation can increase humidity levels in the trailer. An increase in relative humidity (up to 7.5%) has been observed in a sprinkled trailer, which may prevent efficient evaporative cooling (19). A combination of sprinklers and fans can be applied to remove the excessive humidity and cool pigs when the temperature within the vehicle is too high (28). Pereira et al. (72) investigated the combined effects of forced ventilation for 30 min and water misting for 10 m on pigs kept in a trailer vs. a control trailer (not exposed to any cooling system), with both trailers in a stationary position, before unloading at ambient temperatures ranging between 16.5 to 28.1°C. The authors reported that control pigs had a greater need to reduce body temperature by evaporation (as assessed by GTT difference), likely due to heat stress experienced during the wait in the stationary trailer at unloading, whereas treated pigs could maintain their body temperature as they were sufficiently cooled-off by the fan-misting bank during this period.

In winter, the thermal comfort of pigs in the truck can be controlled by partially or fully closing the ventilation openings in order to reduce air-flow (101). Transport Quality Assurance (TQA) guidelines (94) recommend that at temperatures below −12°C trucks should be utilizing 90% boarding (10% side vent opening), and zero boarding above 9.4°C. When the air temperature is below freezing, the boarding is critical to prevent death losses and frostbites on the skin of pigs (1). The use of low boarding level (0–30%) at temperatures below 5°C produced the highest transport losses, while the medium and high boarding level (31 to >61%) appears to have little impact on animal losses at temperatures higher than 5°C (1).

Overall, research findings showed that environmental conditions affect how well pigs are able to cope with transportation and provided evidence about a particular vulnerability to heat stress in market pigs. Under these ambient conditions, the application of cooling systems, i.e., water sprinkling/misting combined or not with fan-assisted ventilation, proved efficient in controlling the microclimate inside the vehicle and providing pigs with better thermal comfort.

## Loading Density

The optimum loading density for pigs during transport involves a trade-off between economic pressure to increase loading density in order to minimize transport costs from a single journey, and the respect of the welfare of animals during transport (2). Loading density specifically refers to the space available to an animal in a truck compartment expressed as kg/m^2^, whereas space allowance is the inverse concept, expressed as m^2^/animal. The EU legislation is based on the evidence that when the loading density is higher than 235 kg/m^2^, not all pigs are able to lie down to rest and cannot rest as they are pushed to continually change their position (3, 69). Lower space allowance has also been associated with increased mortality rates and a higher level of non-ambulatory pigs on arrival at the slaughter plant (30, 102, 103).

Guàrdia et al. (104) also reported a greater incidence of dry, firm, and dark (DFD) pork (+11%) when space allowance was increased from 0.37 to 0.50 m^2^/100 kg, under Spanish commercial transport conditions. Lambooij et al. (69) also found increased muscle pH values at lower space allowances (from 0.66 to 0.33 m^2^/pig), resulting from muscle glycogen depletion at slaughter due to fatigue. This change in muscle physiology likely results from greater physical stress caused by frequent disturbance of lying animals by those seeking a place to rest, and difficulty of standing pigs to maintain balance during vehicle accelerations, braking, and turns (105).

Due to the direct relationship with transport cost, providing too much space is not as common a problem as providing too little. However, providing pigs with too much space may also cause physical stress, as pigs can struggle to maintain their balance due to unexpected movements of the truck, or fighting due to greater freedom to move around in the truck (104, 106). This can lead to muscle fatigue and glycogen depletion, also making pigs prone to produce DFD pork (104). Thus, there is an optimal space allowance, which varies with ambient temperature and pig size (allometrically).

Nannoni et al. (37) reported an increase in transports with at least one DOA when heavier pigs (160 kg) were transported at the EU recommended density of 235 kg/m^2^. For this reason, specific loading densities are recommended for heavier weight pigs because of their different physical and thermal needs (107). Indeed, heavier pigs are more susceptible to heat stress than lighter pigs due to the greater production of body heat (+2% for every additional 5 kg liveweight; (108) and reduced ability to dissipate it (107). According to the latest North American Meat Institute guidelines (109), the minimum recommended truck space required by market weight pigs during winter should increase from 0.40 to 0.46 m^2^/pig as marketing weight increased from 114 kg to 136 kg. In summer, the space increase is from 0.46 to 0.55 m^2^/pig as marketing weight increases from 114 kg to 136 kg. In a series of studies investigating the transportation of pigs, Ritter et al. (20, 103, 110) found that losses were minimized at a floor space of ≥ 0.462 m^2^/pig for pigs weighing 125 kg. Furthermore, at this pig weight, a reduction in floor space from 0.48 to 0.39 m^2^/pig did increase the percentage of fatigued, non-ambulatory pigs, and post-transport plasma CK values (9, 103).

Research has shown that the application of the EU requirement for loading densities should be adjusted according to travel time. Pilcher et al. (111) showed that reducing floor space (from 0.52 to 0.40–0.49 m^2^/100 kg) increased the incidence of fatigued pigs on arrival at the slaughter plant after short transport (<1 h) compared with longer journeys (3 h). However, Guàrdia et al. (96) reported that the application of higher loading densities (0.25 vs. 0.5 m^2^/100 kg) was not detrimental in short journeys (1 h) as it resulted in decreased incidence of PSE pork (indicator of acute stress and muscle acidification due to reduced muscle effort to keep the balance during the vehicle motion) and concluded that, in order to prevent this outcome, the EU-recommended space allowance of 0.425 m^2^/100 kg may be only appropriate for journeys longer than 3 h. These results may be explained by the evidence that giving more space (0.42 and 0.50 vs. 0.35 m^2^/100 kg) does not necessarily result in more pigs lying down, especially during the first 2 h of transport (69, 106), but it causes more disturbance and aggression due to animals being able to move around, loss of balance, and greater risk of being thrown around, and getting stuck and bruised when the vehicle negotiates bends or poor road surfaces (30, 106).

Overall, based on the available scientific evidence, it can be concluded that a loading density for slaughter pigs ≥235 kg/m^2^ does not allow all pigs to lie down and rest at the same time. Furthermore, the impact of loading density varies with ambient conditions, but in general increased loading density increases the risk of a pig becoming non-ambulatory or dying.

## Special Considerations for Young Animals

Most studies measuring the responses of swine to transportation have focused on market weight pigs; however, there is a growing interest and need to understand the effects of transportation on weaned piglets. It has become increasingly common in Canada to transport weaned piglets to specialized growing facilities. This is because sow herds are located in more remote, biosecure regions, and growing pigs are transported to barns that are located in closer proximity to feed production and packing facilities. Like many other species, the combination of weaning stress, along with additional transportation stress can compromise the pigs' welfare, and in extreme cases can lead to death.

Long transportation (8 to > 24 h) of weaned piglets at high ambient temperatures (≥25 up to ≥35°) either can result in a delay in recovery from transport (112) or in a steadily increase in mortality rate, regardless of the exposure to mechanical ventilation and access to drinking water during transport (113).

In a study investigating the effects of transport on 17-day-old piglets, Wamnes et al. (114) found that transportation for <20 min resulted in greater weight loss and slower post-transport weight recovery compared with 6 h transport, likely due to a reduced motivation to feed and drink following transport.

Sutherland et al. (115) tried to determine the required space requirements (0.05, 0.06, and 0.07 m^2^/pig) for weaned pigs during a short (1 h) transportation trip during summer (28.4 ± 1.2°C). The neutrophil to lymphocyte ratio, indicator of the immune response, was greater for piglets transported at 0.05 m^2^/pig compared to 0.06 m^2^/pig and 0.07 m^2^/pig. Piglets transported at 0.05 m^2^ also laid down less during transport. The authors concluded that under the conditions of this study, a minimum space allowance of 0.06 m^2^/pig is preferable for piglet transportation.

Another concern is the greater susceptibility of young animals to cold and heat stress. Brown-Brandl et al. (116) used thermal imagery on pigs between 27 and 37 kg and reported an upper thermal neutral zone of 17.4 to 23.2°C.

Finally, piglet genetics can also have an effect on stress during transport. Averós et al. (117) reported that weaned piglets heterozygous for the stress gene (halothane or HAL^Nn^) were more stressed, based on greater albumin concentrations and total white blood cell and neutrophil counts, compared to those that did not have the halothane allele (HAL^NN^).

In conclusion, the available research findings showed that both short and long transportation have an effect on piglet welfare, with effects being biased by the genetic background and ambient conditions. Weaned pigs are particularly vulnerable to heat stress during transport.

## Concluding Remarks

The research findings presented in this overview allow us to draw clear conclusions on the importance of the proper choice of vehicle. These are based on the distance to travel and of the application of pre-transport fasting and control of environmental conditions on the welfare of pigs during transport and pork quality. Furthermore, it is agreed that the impact of loading density varies with ambient conditions, but in general a greater loading density increases the risk of a pig becoming non-ambulatory or dying. However, the relationship between journey duration and reduced welfare is complex; poor welfare can result from both long and short journeys, resulting in fatigue/dehydration, and acute stress, respectively. A clear conclusion on a maximum transport duration cannot be supported by the current published literature.

The swine industry would therefore benefit from studies investigating factors, such as vehicle design (air-flow patterns, vibration rates, and insulating and cooling systems), loading density (by ambient conditions, travel distance, and pig weight), travel duration, rest/stop duration, and management of pigs during rest stops (either on the truck or in their control post). Most importantly, investigating the associations between these factors would benefit the industry and animals by reducing transport losses, and promoting good animal welfare and meat quality.

The majority of swine transportation studies use market weight pigs, and there is a considerable gap in the scientific literature for newly weaned and for breeding pigs. Understanding how to safely transport cull sows and boars is essential as they present their own set of challenges and risks. Currently almost all cull sows go to one of six assembly yards in Canada and are transported to, and slaughtered in, the USA. Therefore, it would be beneficial to understand the specific challenges faced by cull sows on these long duration journeys. A recent pilot study describing the cull sow market network in the US (involving sows from 21 states and Canada) determined that sows could be in the network for anywhere between 24 to 120 h (max. time under federal law), and the median straight-line distance traveled was 1,057 km prior to harvest (118). However, this was only a pilot study based on sow movements over a 1 week timeframe. Further investigation into larger datasets would be beneficial to the swine industry.

## Author Contributions

FR-L drafted the manuscript, LF supervised the preparation of the manuscript and revised it in collaboration with JB and EB. All authors read and approved the final manuscript.

### Conflict of Interest Statement

The authors declare that the research was conducted in the absence of any commercial or financial relationships that could be construed as a potential conflict of interest.
